# Supplementary material for: A method to determine the duration of the eclipse phase for in vitro infection with a highly pathogenic SHIV strain
Source: Sci Rep. 2015 May 21;5:10371. doi: 10.1038/srep10371 (PMC4440524; doi:10.1038/srep10371)
Supplement: Supporting Information — Supplementary Figures 1-6 [file srep10371-s1.doc]

A method to determine the duration of the eclipse phase for *in vitro* infection with a highly pathogenic SHIV strain

Yusuke Kakizoe^1^, Shinji Nakaoka^2†^, Catherine A. A. Beauchemin^3†^, Satoru Morita^4^, Hiromi Mori^5^, Tatsuhiko Igarashi^5^, Kazuyuki Aihara^6, 7^, Tomoyuki Miura^5*^, Shingo Iwami^1, 8, 9*^

^1^ Department of Biology, Kyushu University, Fukuoka 812-8581, Japan. ^2^ Graduate School of Medicine, The University of Tokyo, Tokyo 113-0033, Japan. ^3^ Department of Physics, Ryerson University, Toronto M5B 2K3, Canada. ^4^ Department of Mathematical and Systems Engineering, Shizuoka University, Shizuoka 432-8561, Japan. ^5^ Institute for Virus Research, Kyoto University, Kyoto 606-8507, Japan. ^6^ Institute of Industrial Science, The University of Tokyo, Tokyo 153-8505, Japan. ^7^ Graduate School of Information Science and Technology, The University of Tokyo, Tokyo 113-8656, Japan. ^8^ PRESTO, JST, Kawaguchi, Saitama 3320012, Japan. ^9^ CREST, JST, Kawaguchi, Saitama 3320012, Japan.

**^*^ Co-corresponding author**

TM: Mailing address: Institute for Virus Research, Kyoto University, Kyoto 606-8507, 53 Shogoin-Kawaracho, Sakyo-ku, Kyoto 606-8507, Japan. Phone: 81-75-751-3984. E-mail: [tmiura@virus.kyoto-u.ac.jp](mailto:tmiura@virus.kyoto-u.ac.jp)

SI: Mailing address: Department of Biology, Faculty of Sciences, Kyushu University, 6-10-1 Hakozaki, Higashi-ku, Fukuoka, Fukuoka 812-8581, Japan. Phone: 81-92-642-2640. E-mail: siwami@kyushu-u.org

^†^ These authors contributed equally to this work

Abstract

The time elapsed between successful cell infection and the start of virus production is called the eclipse phase. Its duration is specific to each virus strain and, along with an effective virus production rate, plays a key role in infection kinetics. How the eclipse phase varies amongst cells infected with the same virus strain and therefore how best to mathematically represent its duration is not clear. Most mathematical models either neglect this phase or assume it is exponentially distributed, such that at least some if not all cells can produce virus immediately upon infection. Biologically, this is unrealistic (one must allow for the translation, transcription, export, etc. to take place), but could be appropriate if the duration of the eclipse phase is negligible on the time-scale of the infection. If it is not, however, ignoring this delay affects the accuracy of the mathematical model, its parameter estimates, and predictions. Here, we introduce a new approach, consisting in a carefully designed experiment and simple analytical expressions, to determine the duration and distribution of the eclipse phase *in vitro*. We find that the eclipse phase of SHIV-KS661 lasts on average one day and is consistent with an Erlang distribution.

Introduction

Mathematical modeling has made important contributions to our quantitative understanding of the course and outcome of viral infections, both *in vitro* and *in vivo*. The analysis of experimental infection data using mathematical models makes it possible to extract information encoded into the observed viral kinetics, and dissect it into the individual parameters driving the infection (e.g., the viral burst size or its clearance rate). These parameter estimates, in turn, can be used to determine the pathogenesis and transmissibility of the virus, predict the course of the disease, and evaluate the effect of antiviral therapy ^1-5^. Virus kinetics can be described using the basic model ^1,6^:


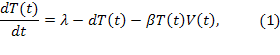


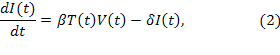


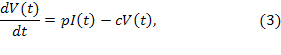


where
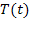
 is the density of uninfected, susceptible target cells which are generated at rate
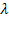
, die at rate
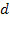
, and become infected at a rate
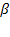
 times the concentration of virus,
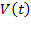
. Once infected,
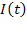
, these cells are assumed to produce virus at constant rate
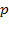
 per cell, until their death which is assumed to occur at an exponential rate of mean duration
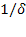
. The virus progeny, produced at a constant rate,
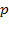
, by infected cells,
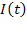
, is lost or cleared exponentially over time at rate
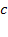
.

This basic model, described by a set of ordinary differential equations (ODEs), implicitly assumes that a newly infected cell can produce and release virus instantly upon infection. In reality, however, there will inevitably always be a delay between the successful infection of a cell and the production of viral progeny, during which time the cell’s internal machinery is hijacked and organized for virus production. This delay is known as the eclipse phase because the input virion disappears (is eclipsed) when its nucleic acid is uncoated shortly after successful entry into the cell, and it will take some time before it is visible again in the form of its output virion progeny. The duration of the eclipse phase depends on a number of intracellular processes related to the synthesis of viral nucleic acid and proteins, viral assembly, maturation, budding, and successful release. The duration of each of these processes, and perhaps more importantly the variability in their duration, has not been studied in details ^7-9^. The mean duration and average time distribution of the eclipse phase vary according to the virus species and even across strains of the same species. For example, the average length of the eclipse phase has been estimated to be around 24 h for infections with the human immunodeficiency virus type 1 (HIV-1) ^10-14^, and varied from 6 h to as much as 12 h for infections with different strains of influenza virus ^4,15-18^.

One simple extension of the basic model is to include an eclipse phase class,
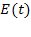
, such that


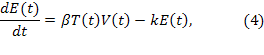


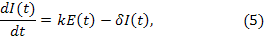


wherein the duration of the eclipse phase is assumed to follow an exponential distribution ^15-17,19^. While this extension does enforce an eclipse delay for at least some of the cells between infection and virus production (i.e., an eclipse phase lasting an average time of
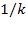
), it still allows some cells to unrealistically begin virus production instantly upon infection. A variety of other, sometimes more realistic, probability distributions for the duration of the eclipse phase, including the Dirac delta, normal, log-normal, gamma, and Erlang distributions, have also been considered ^11,13,14,16-18,20-23^.

The probability distribution for the duration of the eclipse phase corresponds biologically to the variation in the duration of that phase from cell to cell within a culture, with some cells going smoothly through synthesis, assembly and release, while other cells of the same type and within the same culture can take longer in completing certain steps due to the error-prone nature of the various processes involved. The eclipse phase plays an important role in the infection kinetics, exerting most of its influence during early infection events. This is because early in the infection, the viral titers are typically relatively low, and the viral output produced by the first few cells to emerge from the eclipse phase determines the timing of the next round of replication, and the one after that, and so on, contributing directly to the infection growth rate ^4^. The distribution of the eclipse phase, and the variability of the implicit set of mechanisms it stands for, also have important implications for the control of the infection because viral protein production in infected cells is associated with immune recognition and could relate to the establishment of a latent state in some infected cells ^8,18,24,25^. As such, viral kinetics depends strongly on the shape of the probability distribution of the eclipse phase duration: the correct determination of the eclipse phase distribution, and not just its average duration, is of critical importance to viral infection kinetics.

Recently, Petravic *et al.* ^25^ determined that the duration of the eclipse phase for an HIV infection follows a fat-tailed distribution by using an HIV-EGFP reporter virus in a single-cycle (SC) *in vitro* experiment. While this work provides important insights into the detailed intracellular dynamics of HIV infection, it relied experimentally on measurements of EGFP content in infected cells rather than direct measurement of viral proteins, it relied mathematically on an analysis using a non-mechanistic model rather than a more complete kinetic model of infection, and was not further validated through additional, time-course measurements of extracellular viral concentration. Herein, we determined the duration and distribution of the eclipse phase for the infection of HSC-F cells (T lymphocyte cell line) with a highly pathogenic simian/human immunodeficiency virus strain (SHIV-KS661 ^26-29^) *in vitro* ^30^. We were able to directly observe the cells’ distributed transition from the eclipse to the virus-producing infectious phase experimentally by measuring the increasing, cumulative fraction of infected cells which were positive for the Nef SHIV protein. We determined that the eclipse phase in this system lasted on average one day, and varied from cell to cell in a manner consistent with an Erlang distribution. Using our model with an accurate eclipse phase along with extensive infection data, we determined that previous parameter estimates obtained by models which neglect the eclipse phase ^6,29^ overestimated the virus production rate and the duration of the infectious cell lifespan, and underestimated the rate of cell infection by SHIV. The limitations of our findings and future directions for this synergistic approach combining cell culture experiments and mathematical models are also discussed. Although our results rely on SHIV-KS661 and HSC-F cells, our approach for quantitatively understanding of virus dynamics, especially with regards to the eclipse phase distribution, is applicable to a broad range of other virus strains and species.

Results

**Modeling the eclipse phase in virus infection dynamics**

To generalize the basic model and account for the duration of the eclipse phase, we introduce the age of infection,
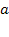
, corresponding to the time elapsed since the successful infection of a cell, i.e. since the start of the eclipse phase (**Figure 1**). Following others, we will refer to cells which have the same age of infection,
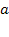
, as a cohort ^31^. Let
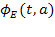
 denote the cohort of cells which have reached age
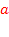
 in the eclipse (non-infectious) phase at present time
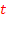
. The population of target and infectious (virus-producing) cells and the virus concentration, at time
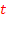
, continue to be represented by
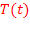
,
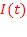
, and
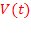
, respectively. We assume that the rate of transition from the eclipse to the infectious phase for a cell that has already spent an age
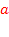
 in the eclipse phase, is given by the hazard rate
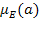
, whose definition ^32^ is such that


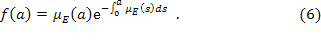


Here,
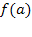
 is a probability density function such that
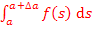
 is the probability that a cell which has already spent an age
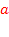
 in the eclipse phase will transition to the infectious phase in the interval of time
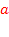
 to
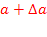
. As such,
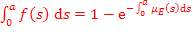
, its associated cumulative distribution function, is the probability that a cell has transitioned to the infectious state by age
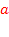
. Its complementary cumulative distribution function,
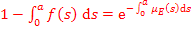
, is the probability that a cell has remained in the eclipse phase at least up to age
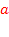
 ^33,34^. Using this framework, the basic model can be extended into an age-structured model with an explicit eclipse phase described by the following partial differential equations (PDEs),


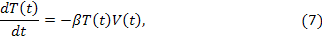


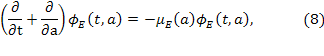


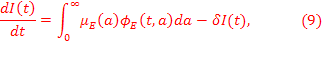


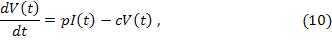


whose boundary condition for
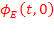
 is given by


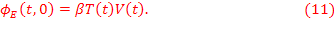


Parameters β, δ, p and c , have the same meaning and dimensions as in the basic model. Because, in our cell culture experiments, the initial cell concentration is close to the carrying capacity of well plates, and target cells replicate slowly, the population of target cells changes very little on the timescale of our experiment (data not shown). We therefore neglected the effects of potential regeneration of target cells in our analysis and in constructing the mathematical model.

We assume the infection is initiated via a virus inoculum,
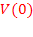
, such that initially all cells are in the uninfected, target state,
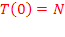
, with no initially infected cells, i.e. no cells in the eclipse, namely
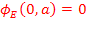
. Consequently, Eqs. (7-10) can be simplified further by the method of characteristics ^35^. That is,
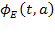
 can be written as


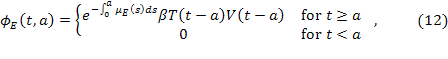


which, when substituted into Eq.(9), simplifies the latter to


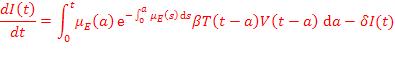


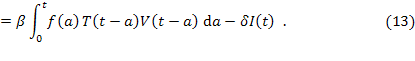


Here the age,
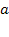
, corresponds to the duration of the eclipse phase, and is distributed according to probability density function
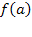
, also called the delay kernel. Thus, the age-structured model Eqs.(7-10) reduces to the above (Eqs. (7), (10), and (13)) delay differential equations (DDEs). Similar mathematical models have been derived in previous studies ^20,23^. Note that if
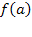
 is an exponential distribution, the DDEs reduces, as expected, to the basic model with an explicit exponentially distributed eclipse phase ^15-17,19^.

**Estimating the distribution and mean duration of the eclipse phase**

To identify the eclipse phase distribution,
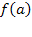
, we carried out a single-cycle (SC) viral yield assay ^17,18,36^. In a SC experiment, cells are infected at a very high multiplicity of infection (MOI), wherein the inoculation consists in several infectious virus per cell. This enables us to reasonably assume that almost all cells are infected simultaneously at the start of the experiment such that
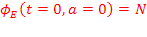
, i.e. 100% of cells are in age zero of the eclipse phase at the start of the infection. Since
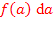
 is the probability that a cell which has reached age
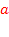
 in the eclipse phase will transition into the infectious phase between age
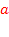
 and
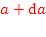
, it follows that
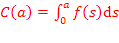
, its associated cumulative distribution function (CDF), corresponds to the fraction of cells which have transitioned out of the eclipse phase and into the infectious phase by age
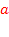
, or by time
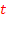
 post-infection since all cells were in age
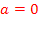
 of the eclipse phase at time
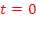
. In this experiment,
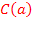
, the CDF for the duration of the eclipse phase, can then be observed as the variation in the times at which the simultaneously infected cells begin virus production. In past work, this has typically been observed indirectly as an increase in the virus yield released into the cell culture medium ^17,18^. Here, we observe this delay in a more direct way by monitoring cells which are positive for a particular virus protein as a marker for the initiation virus production, i.e. transition from the eclipse to the infectious phase. Specifically, we infected HSC-F (monkey CD4+ T cells) with 4.2 TCID_50_/cell of SHIV-KS661, measured the cumulative fraction of cells positive for the Nef SHIV protein, and thus directly observed the CDF of the eclipse phase duration (see **Methods**). Because the Nef protein is synthesized after the integration of SHIV genome into the host genome ^37^, we assume cells expressing the Nef protein are infectious cells which have actively begun virus production and release. We exploited this direct experimental-to-mathematical correspondence,
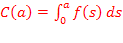
, to evaluate four common candidate probability distribution functions for the true duration of the eclipse phase: the exponential, normal, Weibull, and gamma distributions. The goodness-of-fit and best-fit distribution parameters for each of these four distributions are presented in **Table 1**. The fit of each distribution to the experimental data are shown in **Figure 2**.

Interestingly, an exponential distribution, i.e., the basic model with or without an explicit exponentially distributed eclipse phase ^15-17,19^, yields a very poor fit (highest AIC_C_, see **Methods**) to the experimental data (**Figure 2A**). Furthermore, it estimates a mean value for the duration of the eclipse phase of 1.86 d (days), almost twice the 1 d duration from previous estimates ^10-14^. This overestimation of the eclipse phase duration when assuming it is exponentially-distributed has also been reported in previous work based on indirect observation of the eclipse phase via analysis of viral titer time-course data in SC experiments ^17^.

The remaining three, non-exponential distributions reproduced the experimental data similarly well (**Figure 2BCD**). Additionally, the mean duration of the eclipse phase estimated from all three distributions was consistently around 1 day (**Table 1**), in agreement with previous estimates ^10-14^. Although the very best fit (smallest AIC_C_, see **Methods**) was obtained with the gamma distribution, all three distributions provide an adequate description of the data. Our SC experimental results and statistical analysis indicate that the eclipse phase distribution obeys a non-exponential distribution ^7,25^, consistent with a gamma distributed eclipse phase duration. It is well known that the gamma distribution can reproduce a variety of biological delay distributions ^38^, and for this reason it is commonly used in several mathematical models for virus infection dynamics ^20,21,23^.

**Deriving a simple mathematical model with a realistic eclipse phase**

Hereafter, for our detailed analyses and validations of previous empirical assumption, we choose a gamma distribution as the non-exponential distribution to represent the eclipse phase duration, and investigate SHIV-KS661 infection dynamics. More accurately, for convenience in the remainder of this work, we make use of the Erlang distribution, which is equivalent to the gamma distribution but with the requirement that the shape parameter of the distribution be an integer. The equivalence between the expression for, and the parameters of, the probability density functions of the gamma and Erlang distributions is as follows


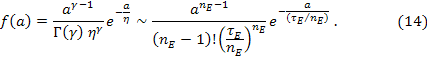


The shape (
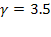
) and scale (
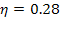
 d) parameters reported in **Table 1** for the gamma distribution correspond to the equivalent shape (
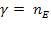
) and scale (
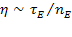
) parameters of the Erlang distribution of mean
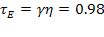
 d (~24 h). Since
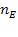
 must be an integer, we chose
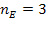
 (
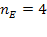
 yields equivalent results, not shown) and uphold
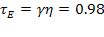
 d such that
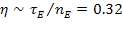
 d. **Figure 3AB** illustrates that the changes in going from the gamma to the Erlang distribution are negligible.

Our choice to use the Erlang distribution over the gamma distribution is motivated by the fact that the former offers the following convenient simplification over the latter. As previously described in ^20,38^, if one defines


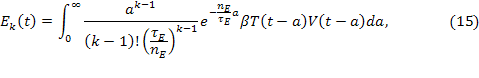


for
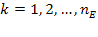
, then integro-differential equation (13) when
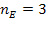
 in the above is equivalent to the following set of ODEs


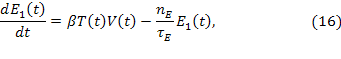


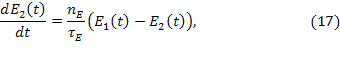


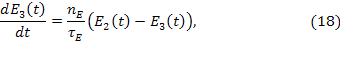


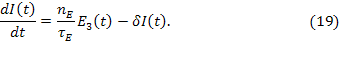


Therefore, the age-structured model Eqs.(7-10) can be replaced with Eqs.(7)(10)(16-19). Similar mathematical models have been empirically proposed in previous studies ^18,21^. The method used to convert our DDEs into ODEs is called the “linear-chain-trick” and is discussed in details elsewhere ^20,38^.

**Analytical expression for infection kinetics in a single-cycle assay**

Interestingly, if one only wishes to reproduce infection kinetics in a SC assay, Eqs.(7)(10)(16-19) can be simplified further by realizing that in a true SC assay, nearly all cells are infected by the initial virus inoculum. Assuming that an MOI of 4.2 is sufficient to infect nearly all cells, we can set
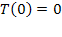
,
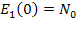
 and
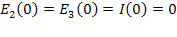
, where
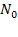
 is the initial cell concentration. Then, Eq.(16) becomes


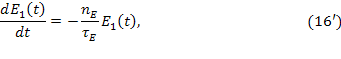


Eqs.(16’)(17-19) decouple from Eq.(7), and the following analytical solution can be found for Eqs.(10)(16’)(17-19):


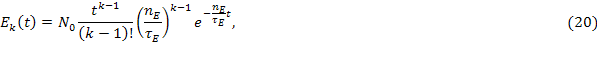


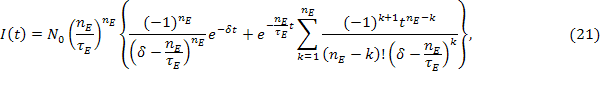


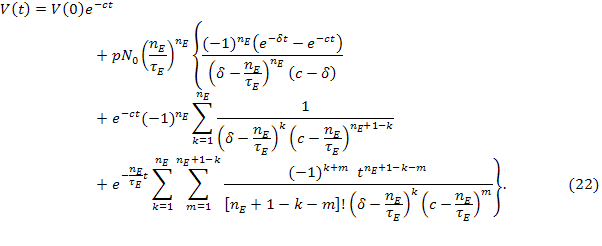


These solutions extend previous analytical approximations of virus infection dynamics ^14,39^, and can be validated through comparison with the experimental data from our SC SHIV infection assay.

We can compare the cumulative fraction of cells positive for the Nef SHIV protein, expressed above as
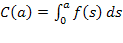
, with that predicted by the analytical model by realizing that it corresponds to the cumulative fraction of cells which, by time
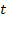
, have transferred into the infectious phase and are now either still infectious and producing virus,
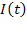
, or have since ceased virus production (i.e., died),
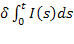
, namely


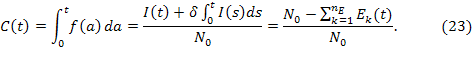


The
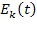
 terms in can be replaced by their analytical solution Eq.(20) such that for , , and we obtain

Note that this does in fact correspond exactly to the cumulative distribution function of the Erlang distribution with shape parameter and scale parameter , illustrated in **Figure 3B**.

Interestingly, we can compare the analytical expression in Eq.(22) for the virus concentration over time, , against actual virus concentration measurements which were sampled alongside the fraction of Nef-positive cells during our SC experiment (see **Methods**). Beyond the eclipse distribution parameters ( and d), the analytical expression for also depends on the virus decay rate (), the rate of virus production by infectious cells (), and the rate of infectious cell death (). These parameters have been estimated previously for the same virus strain in the same cell culture under similar conditions (/d, RNA copies/cell/d, /d) ^6,29^. We substituted these values and our known initial conditions ( RNA copies/ml and cells/ml) into our analytical expressions for and , and present the prediction (not a fit) of Eqs.(21-22) for the viral concentration over time alongside the experimentally measured values in **Figure 3CD**. The significant disagreement between our prediction and the experimental viral load are due to the fact that the parameters used in making our prediction were estimated in previous work from a model which ignored the eclipse phase and assumed newly infected cell could instantly begin producing virus. Perhaps most critically, the value estimated previously for the rate of infectious cell death corresponds to an infectious cell lifespan of ~14 h whereas here we have found that the eclipse phase alone, even prior to cells being infectious and producing virus, lasts ~24 h. By using the old infectious lifespan in combination with the newly determined eclipse phase duration, we are assuming that our infected cells live ~38 h (see **Figure 3C**). Therefore, in **Figure 3CD**, we evaluated different values for the infectious cell lifespan and found that reducing its duration to ~1.7 h (/d) produces a good agreement with the experimental data. This corresponds to a burst size () of 2,300 RNA copies/cell (8-fold less). Naturally, we would expect that the introduction of an Erlang-distributed eclipse phase into the model requires the adjustment of not just how long cells produce virus for (), but also of how much virus they produce per unit time (). We address this point in the next section.

**Analyzing *in vitro* multiple-cycle infection of SHIV-KS661 in HSC-F cells**

In contrast with SC experiments, if infection is initiated with fewer infectious virus than there are cells (an MOI 1 TCID_50_/cell), only a few cells are infected by the inoculum, and these cells go on to infect other cells, leading to successive cycles of infection ^6,16-19,29^. This is called a multiple-cycle (MC) viral yield experiment and is believed to be the typical mode of infection progression for natural virus infections in humans and animals. In addition to the SC experiment introduced above and performed at a MOI of 4.2 TCID_50_ per cell, we have simultaneously carried out SC and MC experiments at four additional MOIs (2.1, 1.05, 0.525 and 0.2625 TCID_50_/cell) for the infection of HSC-F cells with SHIV-KS661, measuring both the total virus yield in the supernatant (RNA copies/ml) and the cumulative fraction of cells positive for the Nef SHIV protein (see **Methods**). Using model Eqs.(7)(10)(16-19), we simultaneously fitted our 80 experimental data points to reproduce these SC and MC experiments and extract the remaining model parameters, namely the virus infectivity (), virus production rate (), and infectious cell lifespan (). The fits were performed as described in the **Methods** section, and are shown in **Figure 4**, with the estimated parameters presented in **Table 2**, and the initial conditions in **Table 3**. The model Eqs.(7)(10)(16-19), reproduces the infection kinetics of both the SC and MC experiments very well.

More interesting, however, is the impact of the introduction of a one day, Erlang-distributed eclipse phase on the extracted parameters. Compared to values estimated previously by fitting similar data to a model without an explicit eclipse phase ^6,29^, our new model estimates a virus production rate ( = 11,000 RNA copies/cell/d versus 22,000 ^29^ and 33,000 ^6^) and an infectious cell lifespan ( = 5.9 h, versus 14 h ^6^ and 20 h ^29^) that are both 2-3 fold smaller than that previously reported. This, in turn, results in an estimated viral burst size ( = 2,700 RNA copies/cell versus 19,000 ^6^ and 22,000 ^29^) 7-8 fold smaller than previous estimates, and consistent with our above estimate (2,300 RNA copies/cell) from predictions made by our analytical expression, Eq.(22). This decrease in the viral burst size is compensated by an equivalent 7-8 fold increase in the estimated virus infectivity ( (RNA/ml・d)^-1^, versus ^6^), such that our estimate for the basic reproductive number ( = 44, versus 41 ^6^) is consistent with previous estimates from models which did not incorporate an eclipse phase.

Discussion

Herein, we investigated the duration and distribution of the eclipse phase for the infection of HSC-F cells (T lymphocytes) with the virulent SHIV-KS661 strain *in vitro*. We directly observed the cells’ distributed transition out of the eclipse phase experimentally by measuring the monotonically increasing, cumulative fraction of cells positive for the Nef SHIV protein, demarking infected cells which have transitioned from the eclipse phase to the virus-producing, infectious phase. Using this data, we evaluated four different candidate distributions for the duration of the eclipse phase: exponential, normal, Weibull, and gamma/Erlang distributions. We found that an exponentially-distributed eclipse phase could not reproduce the experimental SHIV infection data, as others have previously shown for the eclipse phase duration of an influenza infection *in vitro* ^17^. The other three distributions, however, reproduced the experimental data well. This is not surprising if one considers that the eclipse phase duration depends on a sequence of processes. Biologically, each of the processes which make up the eclipse phase has a stochastic duration which follows a particular distribution. Although the number of such processes operating serially might not be very large, the central limit theorem suggests that the sum of their duration, namely the duration of the eclipse phase, should have converged, at least partly, towards a normal distribution whose general shape is also largely consistent with the Weibull and gamma/Erlang distributions. Based on our direct measurements, all three distributions estimated a consistent average duration of one day for the eclipse phase of SHIV infection of HSC-F cells *in vitro*. Although this is the first report based on the direct, experimental measurement of cell transition out of the eclipse phase, our findings regarding the distribution and duration of the eclipse phase are consistent with previous reports ^7,18,20,21,23,25^.

It is interesting to consider how the distribution of the eclipse phase relates to the particular details of virus replication for different viruses. For example, recently, it has been reported that in primary CD4+ T cells, HIV-1 reverse transcription is initiated approximately 3 h post-infection, its integration into the host DNA occurs around 8.5 h after infection, and that all viral transcripts have emerged by 15 h post-infection^7^. It is worth noting that the integration of synthesized HIV-1 DNA into the host genome is a stochastic process, and its distribution obeys a long fat-tailed distribution ^7-9^. Since transcription is generally coupled with translation, the fat-tail characteristic of our distribution for the duration of the SHIV-KS661 eclipse phase is consistent with these longer stochastic delays which one would expect given the known HIV-1 life cycle. In contrast, for viruses such as the influenza virus ^40^ and the hepatitis C virus ^41^, the viral components are reproduced from the viral genome immediately after viral invasion into the host cytoplasm. Consequently, for such viruses, the distributions found for the duration of the eclipse phase are more narrowly distributed and shorter in duration ^4,18^. Thus, the specific life cycle of a virus regulates and explains the distribution and duration of the eclipse phase.

Using a minimal number of reasonable assumptions, we also derived a set of three, independent analytical expressions describing the number of infected cells in the eclipse (Eq.(20)) or infectious phase (Eq.(21)), and the concentration of extracellular virus (Eq.(22)), at any time over the course of a SC virus infection. These expressions extend previously proposed analytical approximations of virus infection dynamics ^14,39^. Using our experimental data, we verified that these novel analytical expressions indeed correctly reproduced the cumulative fraction of cells positive for the Nef SHIV protein. We sought to further validate these expressions by using them to predict our experimentally measured extracellular virus concentration over time. We found that the expressions’ predictions did not reproduce the experimental data well when using parameter estimates from past work wherein a model with no eclipse phase was used ^6,29^. When we accounted for that by allowing the infectious cell lifespan to decrease from that previously estimated, we found that our expressions could indeed faithfully reproduce the experimentally measured virus concentration time-course.

Having demonstrated with our analytical expressions that parameters estimated from mathematical models which do not include an eclipse phase lead to incorrect predictions, we also determined new estimates using more extensive experimental data from five separate virus dilution experiments. Our corrected, full ODE model incorporating our newly derived eclipse phase distribution reproduced all data well, and we identified 3 infection parameters affected by the introduction of the biologically-accurate eclipse phase. Our new estimates for both the virus production rate (SHIV RNA copies/cell/d) and the duration of the infectious cell lifespan (days) were 2-3 fold smaller compared to our previous estimates, leading to an overall 8-fold decrease in the viral burst size (total virus produced by a cell over its infectious lifespan, SHIV RNA copies/cell) ^6,29^. The decrease in these two estimates was countered by a 7-8-fold increase in our estimate of the virus infectivity compare to that previously reported, such that our new estimate for the basic reproductive number () is consistent with previous reports ^11,13,14^. The incorrect estimation of parameters by models which do not faithfully capture the eclipse phase can have important implications for the accuracy of these models’ predictions when used to evaluate antiviral efficacy or relative strain fitness ^14,16,17,20,22,23,42^.

To conclude, we have determined that failure to properly account for the duration and distribution of the eclipse phase will lead to incorrect estimates of key viral replication parameters, affecting also the accuracy of any work derived from the incorrectly parameterized models. To remedy this situation, we have introduced a set of three independent analytical expressions which accurately capture the fraction of infected cells in the eclipse and infectious phases, and the extracellular virus concentration at any time over the course of a single-cycle virus infection *in vitro*. We believe that, along with a carefully designed *in vitro* experimental system like that described herein, these three expressions constitute a unique and invaluable tool for characterizing the distribution and mean duration of the eclipse phase in various virus strains and species. Importantly, these analytical expressions are relatively general, and should be applicable to a variety of SC virus infection experiments. The only foreseeable challenge in applying these approaches more generally to other viral strains or species would be the identification of a virus protein (like the Nef SHIV protein used herein) which can act as an appropriate marker for the transition of cells from the eclipse to the infectious phase. The experimental-mathematical approach adopted here has quantitatively revealed the replication dynamics of retroviruses ^6,7,25,29^ and other viruses ^16,18,19^ in cell culture systems. A data-driven mathematical approach can elucidate viral infection dynamics in ways that are impossible by conventional experimental strategies alone.

Methods

**Viruses and cell culture**

The virus stock of SHIV-KS661 ^43^ was prepared in a CD4+ human T lymphoid cell line, M8166 (a subclone of C8166) ^44^ and concentrated using Amicon ultra-4 centrifugal filter devices (UFC810024; Merck Millipore Ltd., Tullagreen/Carrigtwohill/Co. Cork, Ireland). The stock was sterilized by 0.45 µm filtration and stored in liquid nitrogen until use. Establishment of the HSC-F cell line has been previously described in ^30^. This is a cynomolgous monkey CD4+ T-cell line from fetal splenocyte that was immortalized by infection with Herpesvirus saimiri subtype C. The cells were cultured in RPMI-1640 medium supplemented with 10% fetal calf serum at 37˚C and 5% CO2 in humidified condition.

***In vitro* experiment**

HSC-F cells were inoculated in 1.5 ml micro centrifugation tube at different MOIs (4.2, 2.1, 1.05, 0.525 and 0.2625; MOI = TCID50/cell) of SHIV-KS661 and centrifuged at 4,000 rpm for 1 h at 25˚C. After the inoculation, cells were washed three times to remove the infection medium and suspended in 850 µl of fresh medium and divided to four wells (210 µl per well and an initial cell concentration of 1.2×10^6^ cells/ml in each well) of a 96 well plate and cultured. They were used for the measurement one by one in turn. This experiment was performed in dividing into twice. One experiment was measurement for 8, 12, 16 and 20 hours after inoculation and another experiment was measurement for 24, 28, 32 and 73 hours after inoculation. At each measuring point, 50 µl of the culture supernatant of one well was harvested. Harvested culture supernatants were frozen and stored at -80˚C until they were assayed via RT-PCR as described below. The remaining cells were re-suspended after addition of 50 µl of fresh medium and used for cell count and FACS analysis.

**Quantification of viable and infected cells**

Virus infection of the HSC-F cells was measured by FACS analysis using markers for surface CD4 and intracellular SIV Nef antigen expression. The number of total and viable cells were first determined using an automated blood cell counter (F-820; Sysmex, Kobe, Japan). Viable HSC-F cells (gated by forward- and side-scatter results) were examined by flow cytometry to measure the surface CD4 and intracellular SIV Nef antigen expression. Cells were permeabilized with detergent-containing buffer (Permeabilizing Solution 2, BD Biosciences, San Jose, CA). The permeabilized cells were stained with phycoerythrin conjugated anti-human CD4 monoclonal antibody (Clone Nu-TH/I; Nichirei, Tokyo, Japan) and anti-SIV Nef monoclonal antibody (04-001, Santa Cruz Biotechnology, Santa Cruz, CA) labeled by Zenon Alexa Fluor 488 (Invitrogen, Carlsbad, CA), and analyzed on FACSCalibur (BD Biosciences, San Jose, CA).

**Quantification of viral load**

We followed the kinetics of the total SHIV-KS661 viral load. The total viral load was measured with a real-time PCR quantification assay, as described previously ^6,29^. Briefly, total RNA was isolated from the 100 fold diluted culture supernatants (140 µl) of virus-infected HSC-F cells with a QIAamp Viral RNA Mini kit (QIAGEN, Hilden, Germany). RT reactions and PCR were performed by a QuantiTect probe RT-PCR Kit (QIAGEN, Hilden, Germany) using the following primers for the gag region; SIV2-696F (5'-GGA AAT TAC CCA GTA CAA CAA ATAGG-3') and SIV2-784R (5'-TCT ATC AAT TTT ACC CAGGCA TTT A-3'). A labeled probe, SIV2-731T (5'-Fam-TGTCCA CCT GCC ATT AAG CCC G-Tamra-3'), was used for detection of the PCR products. These reactions were performed with a Prism 7500 Sequence Detector (Applied Biosystems, Foster City, CA) and analyzed using the manufacturer’s software. For each run, a standard curve was generated from dilutions whose copy numbers were known, and the RNA in the culture supernatant samples was quantified based on the standard curve.

**Comparison of the goodness-of-fit for eclipse phase distributions**

The cumulative fraction of cells positive for the Nef SHIV protein was collected at eight different times post-infection (8 h, 12 h, 16 h, 20 h, 24 h, 28 h, 32 h, 73 h) over the course of infection of HSC-F cells initiated with SHIV-KS661 inocula at an MOI of 4.2 TCID_50_/cell. For each of our four candidate probability distributions functions for the duration of the eclipse phase (Exponential, Weibull, Normal, Gamma), we performed a fit of their associated distributions, , to our experimental data, , using the Mathematica function FindMinimum to minimize the following objective function

where is the cumulative distribution function corresponding to one of our four candidate probability distribution functions (i.e., either the exponential, normal, Weibull, or gamma distribution; see **Table 1**) and is the measured cumulative fraction of infectious (virus-producing) cells (i.e. cells positive for the Nef SHIV protein) at the experimental sampling time, .

To quantify the goodness-of-fit between the distributions for the eclipse phase duration and the experimental SC data for the cumulative fraction of cells positive for the Nef virus protein, we calculated the second-order Akaike’s “an information criterion” () for each fit using

where is the number of parameters of each probability distribution ( for the exponential distribution, and for the other distributions considered), is the number of data points (i.e., ), and is the sum of squared residuals between the experimental data and the best-fitted CDF of each probability distribution ^16^.

**Identification of best-fit parameters from single- and multiple-cycle data**

The total virus concentration in the supernatant and the cumulative fraction of cells positive for the Nef SHIV protein were collected at eight different times post-infection (8 h, 12 h, 16 h, 20 h, 24 h, 28 h, 32 h, 73 h) over the course of five separate infections of HSC-F cells initiated with decreasing SHIV-KS661 inocula (MOI of 4.2, 2.1, 1.05, 0.525 and 0.2625 TCID_50_/cell). A nonlinear least-square fit was performed simultaneously against all experimental data using the Mathematica function FindMinimum to minimize the following objective function:

where

is the cumulative fraction of infectious (virus-producing) cells (i.e. cells positive for the Nef SHIV protein) and the SHIV concentration in the supernatant (RNA copies/ml) at the experimental sampling time, . Index corresponds to one of the five experiments performed at a given MOI, and superscripts “eqn” and “dat” designate data points that were either generated from the model Eqs.(7)(10)(16-19) or measured experimentally, respectively.

Model (7)(10)(16-19) has a total of 6 parameters (, , , , , ) which will be shared by the 5 different experiments. We fix d and as these have been established already from the MOI = 4.2 TCID_50_/cell experiment, and fix /d as determined in previous work ^6,29^. There are also 30 different initial conditions, i.e. 6 per MOI experiment (, , , , , ), which we reduce by setting , and , where cells/ml is the initial cell concentration, and is the fraction of cells successfully infected by the inoculum MOI by the end of the 1 h incubation period. This leaves a total of 13 quantities (, , , , ) to be estimated from our 80 experimental measurements.

References

1 Perelson, A. S. Modelling viral and immune system dynamics. *Nat Rev Immunol* **2**, 28-36, doi:10.1038/nri700 (2002).

2 Simon, V. & Ho, D. D. HIV-1 dynamics in vivo: implications for therapy. *Nat Rev Microbiol* **1**, 181-190, doi:10.1038/nrmicro772 (2003).

3 Guedj, J., Rong, L., Dahari, H. & Perelson, A. S. A perspective on modelling hepatitis C virus infection. *J Viral Hepat* **17**, 825-833, doi:10.1111/j.1365-2893.2010.01348.x (2010).

4 Beauchemin, C. A. & Handel, A. A review of mathematical models of influenza A infections within a host or cell culture: lessons learned and challenges ahead. *BMC Public Health* **11 Suppl 1**, S7, doi:10.1186/1471-2458-11-s1-s7 (2011).

5 Iwami, S., Koizumi, Y., Ikeda, H. & Kakizoe, Y. Quantification of viral infection dynamics in animal experiments. *Front Microbiol* **4**, 264, doi:10.3389/fmicb.2013.00264 (2013).

6 Iwami, S. *et al.* Identifying viral parameters from in vitro cell cultures. *Front Microbiol* **3**, 319, doi:10.3389/fmicb.2012.00319 (2012).

7 Mohammadi, P. *et al.* 24 hours in the life of HIV-1 in a T cell line. *PLoS Pathog* **9**, e1003161, doi:10.1371/journal.ppat.1003161 (2013).

8 Mohammadi, P. *et al.* Dynamics of HIV latency and reactivation in a primary CD4+ T cell model. *PLoS Pathog* **10**, e1004156, doi:10.1371/journal.ppat.1004156 (2014).

9 Weinberger, L. S., Burnett, J. C., Toettcher, J. E., Arkin, A. P. & Schaffer, D. V. Stochastic gene expression in a lentiviral positive-feedback loop: HIV-1 Tat fluctuations drive phenotypic diversity. *Cell* **122**, 169-182, doi:10.1016/j.cell.2005.06.006 (2005).

10 Kim, S. Y., Byrn, R., Groopman, J. & Baltimore, D. Temporal aspects of DNA and RNA synthesis during human immunodeficiency virus infection: evidence for differential gene expression. *J Virol* **63**, 3708-3713 (1989).

11 Little, S. J., McLean, A. R., Spina, C. A., Richman, D. D. & Havlir, D. V. Viral dynamics of acute HIV-1 infection. *J Exp Med* **190**, 841-850 (1999).

12 Dixit, N. M., Markowitz, M., Ho, D. D. & Perelson, A. S. Estimates of intracellular delay and average drug efficacy from viral load data of HIV-infected individuals under antiretroviral therapy. *Antivir Ther* **9**, 237-246 (2004).

13 Ribeiro, R. M. *et al.* Estimation of the initial viral growth rate and basic reproductive number during acute HIV-1 infection. *J Virol* **84**, 6096-6102, doi:10.1128/jvi.00127-10 (2010).

14 Nowak, M. A. *et al.* Viral dynamics of primary viremia and antiretroviral therapy in simian immunodeficiency virus infection. *J Virol* **71**, 7518-7525 (1997).

15 Baccam, P., Beauchemin, C., Macken, C. A., Hayden, F. G. & Perelson, A. S. Kinetics of influenza A virus infection in humans. *J Virol* **80**, 7590-7599, doi:10.1128/jvi.01623-05 (2006).

16 Beauchemin, C. A. *et al.* Modeling amantadine treatment of influenza A virus in vitro. *J Theor Biol* **254**, 439-451, doi:10.1016/j.jtbi.2008.05.031 (2008).

17 Holder, B. P. & Beauchemin, C. A. Exploring the effect of biological delays in kinetic models of influenza within a host or cell culture. *BMC Public Health* **11 Suppl 1**, S10, doi:10.1186/1471-2458-11-s1-s10 (2011).

18 Pinilla, L. T., Holder, B. P., Abed, Y., Boivin, G. & Beauchemin, C. A. The H275Y neuraminidase mutation of the pandemic A/H1N1 influenza virus lengthens the eclipse phase and reduces viral output of infected cells, potentially compromising fitness in ferrets. *J Virol* **86**, 10651-10660, doi:10.1128/jvi.07244-11 (2012).

19 Fukuhara, M. *et al.* Quantification of the dynamics of enterovirus 71 infection by experimental-mathematical investigation. *J Virol* **87**, 701-705, doi:10.1128/jvi.01453-12 (2013).

20 Mittler, J. E., Sulzer, B., Neumann, A. U. & Perelson, A. S. Influence of delayed viral production on viral dynamics in HIV-1 infected patients. *Math Biosci* **152**, 143-163 (1998).

21 Grossman, Z. *et al.* Ongoing HIV dissemination during HAART. *Nat Med* **5**, 1099-1104, doi:10.1038/13410 (1999).

22 Nelson, P. W., Murray, J. D. & Perelson, A. S. A model of HIV-1 pathogenesis that includes an intracellular delay. *Math Biosci* **163**, 201-215 (2000).

23 Nelson, P. W. & Perelson, A. S. Mathematical analysis of delay differential equation models of HIV-1 infection. *Math Biosci* **179**, 73-94 (2002).

24 Jordan, A., Defechereux, P. & Verdin, E. The site of HIV-1 integration in the human genome determines basal transcriptional activity and response to Tat transactivation. *Embo j* **20**, 1726-1738, doi:10.1093/emboj/20.7.1726 (2001).

25 Petravic, J. *et al.* Intracellular dynamics of HIV infection. *J Virol* **88**, 1113-1124, doi:10.1128/jvi.02038-13 (2014).

26 Kozyrev, I. L. *et al.* Characterization of less pathogenic infectious molecular clones derived from acute-pathogenic SHIV-89.6p stock virus. *Virology* **282**, 6-13, doi:10.1006/viro.2000.0839 (2001).

27 Miyake, A. *et al.* Rapid dissemination of a pathogenic simian/human immunodeficiency virus to systemic organs and active replication in lymphoid tissues following intrarectal infection. *J Gen Virol* **87**, 1311-1320, doi:10.1099/vir.0.81307-0 (2006).

28 Matsuda, K. *et al.* In vivo analysis of a new R5 tropic SHIV generated from the highly pathogenic SHIV-KS661, a derivative of SHIV-89.6. *Virology* **399**, 134-143, doi:10.1016/j.virol.2010.01.008 (2010).

29 Iwami, S. *et al.* Quantification system for the viral dynamics of a highly pathogenic simian/human immunodeficiency virus based on an in vitro experiment and a mathematical model. *Retrovirology* **9**, 18, doi:10.1186/1742-4690-9-18 (2012).

30 Akari, H. *et al.* In vitro immortalization of Old World monkey T lymphocytes with Herpesvirus saimiri: its susceptibility to infection with simian immunodeficiency viruses. *Virology* **218**, 382-388, doi:10.1006/viro.1996.0207 (1996).

31 Iannelli, M. Mathematical theory of age-structured population dynamics. *Giardini Editori e Stampatori in Pisa* (1995).

32 Cox, D. R. & Oakes, D. *Analysis of survival data*. Vol. 21 (CRC Press, 1984).

33 Rong, L., Feng, Z. & Perelson, A. S. Mathematical analysis of age-structured HIV-1 dynamics with combination antiretroviral therapy. *SIAM Journal on Applied Mathematics* **67**, 731-756 (2007).

34 Rong, L. *et al.* Analysis of hepatitis C virus decline during treatment with the protease inhibitor danoprevir using a multiscale model. *PLoS computational biology* **9**, e1002959, doi:10.1371/journal.pcbi.1002959 (2013).

35 Metz, J. A. & Diekmann, O. The dynamics of physiologically structured populations. *Lecture notes in biomathematics* **68** (1986).

36 Gaush, C. R. & Smith, T. F. Replication and plaque assay of influenza virus in an established line of canine kidney cells. *Appl Microbiol* **16**, 588-594 (1968).

37 Freed, E. O. HIV-1 replication. *Somat Cell Mol Genet* **26**, 13-33 (2001).

38 MacDonald, N. & MacDonald, N. *Biological delay systems: linear stability theory*. (Cambridge University Press, 2008).

39 Smith, A. M., Adler, F. R. & Perelson, A. S. An accurate two-phase approximate solution to an acute viral infection model. *J Math Biol* **60**, 711-726, doi:10.1007/s00285-009-0281-8 (2010).

40 Watanabe, T., Watanabe, S. & Kawaoka, Y. Cellular networks involved in the influenza virus life cycle. *Cell host & microbe* **7**, 427-439, doi:10.1016/j.chom.2010.05.008 (2010).

41 Miyanari, Y. *et al.* The lipid droplet is an important organelle for hepatitis C virus production. *Nature cell biology* **9**, 1089-1097, doi:10.1038/ncb1631 (2007).

42 Ciupe, M. S., Bivort, B. L., Bortz, D. M. & Nelson, P. W. Estimating kinetic parameters from HIV primary infection data through the eyes of three different mathematical models. *Math Biosci* **200**, 1-27, doi:10.1016/j.mbs.2005.12.006 (2006).

43 Shinohara, K. *et al.* A highly pathogenic simian/human immunodeficiency virus with genetic changes in cynomolgus monkey. *J Gen Virol* **80 ( Pt 5)**, 1231-1240 (1999).

44 Clapham, P. R. *et al.* Human immunodeficiency virus infection of monocytic and T-lymphocytic cells: receptor modulation and differentiation induced by phorbol ester. *Virology* **158**, 44-51 (1987).

Acknowledgments

We thank Yoh Iwasa, Kei Sato, Hisashi Inaba, Yasuhiro Takeuchi, Yoshihisa Morita and Hiroshi Nishiura for many helpful comments on this manuscript. This research was supported in part by the Kyushu University Fund (to Y.K.), Grants-in-Aid for Young Scientists B25871132 (to S.N.) and B25800092 (to S.I.) from the Japan Society for the Promotion of Science (JSPS), the Commissioned Research program of the Ministry of Health, Labour and Welfare, Japan (to S.N., K.A., and S.I. H26-ShinkoJitsuyoka-General-016), Kyushu University Short-term Young Scholar Exchange Program, which is based on Japanese Ministry of Education, Culture, Sports, Science and Technology’s grant The Program for Promoting the Enhancement of Research Universities (to C.A.A.B., and S.I.), Discovery Grant from the Natural Sciences and Engineering Research Council of Canada (to C.A.A.B.), Early Researcher Award (to C.A.A.B) from the Ministry of Research and Innovation of the Government of Ontario, JST CREST program (to S.M., and S.I.), the Aihara Innovative Mathematical Modeling Project, JSPS, through the “Funding Program for World-Leading Innovative R & D on Science and Technology (FIRST Program)”, initiated by Council for Science and Technology Policy (to K.A., and S.I.), JST PRESTO program (to S.I.), JSPS KAKENHI Grant Number 10192783 (to S.I.), with additional funding from the Inamori Foundation (to S.I.).

Author Contributions

Conceived and designed the experiments: TI TM SI. Performed the experiments: HM TI TM. Analyzed the data: YK CAAB SI. Contributed reagents/materials/analysis tool: HM TI TM. Wrote the paper: YK SN CAAB SM TI KA TM SI. Developed the modeling framework: YK SN CAAB SM KA SI.

Competing Interests

The authors have declared that no competing interests exist.

Figure legends

**Figure 1. A schematic representation of the mathematical model.** After a virion, , successfully enters and infects a susceptible target cell, , at infection rate, , the newly infected cell progresses through different stages of cell populations, , which are structured according to the time elapsed, , since virus entry. Each of these stages has a corresponding age-dependent hazard rate, , for the probability that the newly infected cell in the eclipse phase transitions to the infectious state (i.e., becomes infectious, ) and begins virus production. An infectious, virus-producing cell, , produces progeny virions at constant rate , and dies at rate . The virions are cleared at rate .

**Figure 2. Fits of several probability distributions to a single-cycle viral yield experiment.** During the SC experiments at an MOI of 4.2 TCID_50_/cell, the ratio of Nef positive, infectious, virus-producing cells to total cells was measured over time. The symbols denote experimental time course data and the solid line displays the best fit of the cumulative distribution function for the (A) exponential, (B) normal, (C) Weibull and (D) gamma distributions to the experimental data. The overlaid (E) cumulative distribution functions and their associated (F) probability density functions are also shown for comparison.

**Figure 3. Reconstruction of the single-cycle viral yield experiment.** The (A) probability density function and (B) cumulative distribution function for our gamma distribution with a shape parameter of 3.5 and our Erlang distribution with a shape parameter of 3, both with a mean of 0.98 days are shown side-by-side for comparison. Prediction of the (C) fraction of infectious cells, , and (D) extracellular viral load, , from the analytical expressions, Eqs. (21-22). Using a typical model with no eclipse phase (no ) and a previously estimated infectious cell death rate of /d results in an incorrect prediction for the viral load time course. Using this same infectious cell death rate in Eqs.(21-22) results a much larger fraction of cells infected and appearing much later compared to the model without an eclipse phase, and overestimates the viral load. Adjusting the infectious cell death rate to /d results in fewer infected cells peaking earlier, and agrees well with the experimental viral load.

**Figure 4. Fits of mathematical model to single- and multiple-cycle viral yield experiment.** During the SC and MC experiments at five different MOIs (TCID_50_/cell), the ratio of virus-producing cells to total cells and the amount of extracellular viral RNA in the supernatant were measured. The symbols denote the ratio of virus-producing cells in (A) and viral load in (B), respectively, and the solid lines are the best fit of the mathematical model, Eqs.(7)(10)(16-19), to the data.

**Table 1. Estimated parameter values of the probability distribution functions**

| Probability distribution | Cumulative distribution function | Parameters | | Mean | SSR | AIC_C_ |
| --- | --- | --- | --- | --- | --- | --- |
| Exponential |  | †1 | | 1.86 d | 1.34 | -7.89 |
|  |  | 0.54 d^-1^ | |  |  |  |
| Normal |  | †2 | †3 | 0.89 d | 0.26 | -15.4 |
|  |  | 0.89 d | 0.40 d |  |  |  |
| Weibull |  | †4 | †5 | 0.96 d | 0.28 | -14.8 |
|  |  | 2.1 | 1.08 d |  |  |  |
| Gamma |  |  |  | 0.98 d | 0.25 | -15.9 |
|  |  | 3.5 | 0.28 d |  |  |  |

^†1^ Scale parameter (units d = days), ^†2^ Mean, ^†3^ Standard deviation, ^†4^ Shape parameter, ^†5^ Scale parameter

**Table2. Parameters values and derived quantities for the single- and multiple-cycle viral yield experiment**

| Parameter Name | Symbol | Unit | Value |
| --- | --- | --- | --- |
| Parameters obtained from simultaneous fit to full *in vitro* dataset | | | |
| Rate constant for infection |  | (RNA/ml・day)^-1^ | 6.40×10^-10^ |
| Death rate of virus-producing cells |  | day^-1^ | 4.09 |
| Production rate of total virus |  | RNA copies/cell・day^-1^ | 1.10×10^4^ |
| Quantities derived from fitted values | | | |
| Half-life of virus producing cells |  | days | 0.17 |
| Viral burst size |  | RNA copies/cell | 2.68×10^3^ |
| Basic reproductive number of virus |  | ― | 44.0 |

**Table 3. Initial values for the single- and multiple-cycle viral yield experiment**

| Variable | Unit | Fitted initial conditions at experimental MOIs (TCID_50_/cell) of | | | | |
| --- | --- | --- | --- | --- | --- | --- |
|  |  | 4.2 | 2.1 | 1.05 | 0.525 | 0.2625 |
|  | cells/ml | 2.58 | 187.6 | 2.69×10^3^ | 2.11×10^5^ | 5.77×10^5^ |
|  | cells/ml | 9.99×10^5^ | 9.99×10^5^ | 9.73×10^5^ | 7.89×10^5^ | 4.23×10^5^ |
|  | RNA copies/ml | 1.64×10^9^ | 1.09×10^9^ | 7.55×10^8^ | 1.98×10^8^ | 7.02×10^7^ |
